# Explore the Role of the rs1801133-PPARG Pathway in the H-type Hypertension

**DOI:** 10.1155/2022/2054876

**Published:** 2022-03-20

**Authors:** Xiuwen Liang, Tingting He, Lihong Gao, Libo Wei, Di Rong, Yu Zhang, Yu Liu

**Affiliations:** ^1^Cardiology Department, Hulunbeir China Mongolia Hospital Affiliated to the Teaching Hospital of Inner Mongolia Medical University, No. 58 West Street, Hailar District, Hulunbuir, Inner Mongolia 021000, China; ^2^Cardiology Department, Hulunbeir People's Hospital, No. 20, Shengli Street, Hailar District, Hulunbuir, Inner Mongolia 021008, China; ^3^Neurology Department, Hulunbeir People's Hospital, No. 20, Shengli Street, Hailar District, Hulunbuir, Inner Mongolia 021008, China; ^4^Cardiology Department, Inner Mongolia Medical University, No. 5 Xinhua Street, Huimin District, Hohhot, Inner Mongolia 010110, China; ^5^Geriatric Department, Hulunbeir People's Hospital, No. 20, Shengli Street, Hailar District, Hulunbuir, Inner Mongolia 021008, China; ^6^Cardiology Department, Inner Mongolia Minzu University, No. 536, West Huolinhe Street, Tongliao, Inner Mongolia 028000, China

## Abstract

Both rs1801133 mutation on Methylenetetrahydrofolate reductase (MTHFR) gene and transcription factor peroxisome proliferator-activated gamma (PPARG) have been associated with plasma homocysteine (Hcy) levels and hypertension. However, their role in H-type hypertension remains unclear. In this study, we first tested the association between rs1801133 genotypes and Hcy level in H-type hypertension using clinical profiles collected from 203 patients before and after the treatment using enalapril maleate and folic acid tablets (EMFAT). Then, we constructed a literature-based pathway analysis to explore the role of the rs1801133-PPARG signaling pathway in H-type hypertension and its treatment. Although presented similar blood pressure, the patients with TT genotype of rs1801133 were much younger (*p* value <0.05) and significantly higher in Hcy levels (*x*^2^ = 6.11 and *p* < 0.005) than that in the CC and CT genotype groups. Pathway analysis showed that T-allele of rs1801133 could inhibit the expression of PPARG through the downregulation of folate levels and upregulation of Hcy levels, which increased the risk of hypertension and hyperhomocysteinemia. Treatment using EMFAT led to similarly decreased Hcy levels for all patients with different genotypes (*x*^2^ = 86.00; *p* < 0.36), which may occur partially through the activation of PPARG. Moreover, even after treatment, the patients with TT genotype still presented significantly higher Hcy levels (*x*^2^ = 7.87 and *p* < 0.001). Our results supported that rs1801133 mutation could play a role in H-type hypertension, which might be partially through the downregulation of PPARG. Moreover, PPARG might also be involved in treating H-type hypertension using EMFAT.

## 1. Introduction

H-type hypertension is essential hypertension accompanied by an elevated level of plasma homocysteine (Hcy) levels (≥10 *μ*mol/L) [1]. Hcy levels typically are higher in men than in women and increase with age [2, 3]. Elevated levels of Hcy have been correlated with the occurrence of many disorders [4–6], including blood clotting and H-type hypertension [7, 8]. In China, the prevalence of hypertension is 29.6% [9], and about 75% of hypertensive patients present elevated homocysteine levels [10].

Hcy is a sulfur-containing amino acid derived from the metabolism of methionine and is metabolized by one of the two pathways: remethylation or transsulfuration. Methylenetetrahydrofolate reductase (MTHFR) is a key enzyme of homocysteine metabolism, providing a methyl group for Hcy remethylation into methionine and maintaining the normal levels of Hcy in the body [11]. Mutations of the MTHFR gene decrease MTHFR enzyme activity that prevents Hcy remethylation and increases Hcy levels in plasma [11, 12]. The MTHFR gene has at least two functional polymorphisms, 677T and 1298C. The MTHFR C677T (rs1801133) mutation is relatively common, which increases the risk of high Hcy levels [13].

Multiple studies have been conducted on the association between rs1801133 polymorphism and H-type hypertension. However, the results obtained remained controversial. For example, one recent clinical study involving 241 cases found a significant higher TT genotype prevalence in H-type hypertension patients (Hcy ≥ 15 *μ*mol/L) than that in non-H-type hypertension patients (Hcy < 15 *μ*mol/L), suggesting that TT variant of rs1801133 could be associated with H-type hypertension [11]. However, another recent study involving 185 patients found no association between the rs1801133 polymorphism and H-type hypertensive [8].

Peroxisome proliferator-activated receptor gamma (PPARG) is a type II nuclear receptor (protein regulating genes) encoded by the PPARG gene [14]. Many studies confirmed that PPARG plays a protective role in hypertension [15, 16]. Several studies also suggested that increased activity of PPARG could lower plasma Hcy [17], while hyperhomocysteinemia has been found to reduce the protein expression of PPARG [18, 19]. However, no study has reported a direct relationship between PPARG and H-type hypertension so far.

Here, we integrated clinical data analysis and large-scale literature-based pathway analysis to study the potential relation between PPARG and rs1801133 mutation and their potential role in the pathogenesis and treatment of H-type hypertension. Our results indicated that the rs1801133-PARG signaling pathway might be associated with H-type hypertension and play roles in its treatment using enalapril maleate and folic acid tablets (EMFAT).

## 2. Materials and Methods

The rest of this study was organized as follows. We first examined the relationship between different rs1801133 genotypes and plasma Hcy levels in H-type hypertension using clinical profiles collected from 203 patients. The treatment effect using EMFAT was also studied. Then, we conducted large-scale literature-based pathway analysis to construct signaling pathways and explore the relationship between rs1801133 mutation, PPARG, and H-type hypertension.

### 2.1. Clinical Data Collection

Clinical data were collected from newly diagnosed H-type hypertension patients diagnosed in Hulunbuir People's Hospital from October 2017 to October 2018. All patients met the standards of the China Guidelines for Prevention and Treatment of Hypertension (2010) with plasma Hcy ≥ 10 *μ*mol/L. The inclusion criteria are as follows: the patients met the diagnostic criteria of type H hypertension. The Ethics Committee of HulunBuir Hospital approved the study, and all participants provided consent to participate after being informed of the study protocol. The exclusion criteria are as follows: all subjects with the following conditions were excluded, including secondary hypertension, malignant tumors, immune diseases, liver and kidney dysfunction (liver enzymes: 3 times higher than the normal range, blood creatinine > 256 *μ*mol/L), and those who were taking the medications that may affect the levels of plasma Hcy within six months, such as folic acid, vitamins, methotrexate, and oral contraceptives.

In total, 203 patients passed the recruiting criteria and were included in this study. According to their MTHFR genotype, these 203 H-type hypertension patients were divided into three groups: CC (wild-type homozygous) group, CT (heterozygous) group, and TT (mutant allele homozygous) group. The basic demographic information of patients was recorded, including gender, age, and ethnicity. In addition, the clinical parameters were recorded and compared among groups, including Hcy, blood pressure (systolic blood pressure and diastolic blood pressure), blood lipids (including triglyceride TG and cholesterol TC), fasting blood glucose value, and carotid artery color Doppler ultrasound. All the patients were treated with oral EMFAT for three months, with the Hcy level measured before and after the treatment.

### 2.2. MTHFR Gene Determination

The polymerase chain amplification (PCR) and the MX3005p fluorescently labeled probes were used to detect the genotype of the 677C/T polymorphic sites of the MTHFR gene in clinical specimens. The whole gene DNA of the blood sample was extracted and detected by fluorescent PCR technology. The genotype of MTHFR in the sample was distinguished by the difference in fluorescence.

### 2.3. Determination of Plasma Hcy

The fasting venous blood was collected from patients. An enzymatic cycling assay for homocysteine was evaluated using a Roche Modular Analytics P800 automatic chemistry analyzer. Plasma Hcy was evaluated using a Roche Modular Analytics P800 chemistry analyzer. The kit was provided by Oursa Company.

### 2.4. Statistical Analysis

SPSS21.0 software was used for statistical analysis. The measurement data were described by the mean ± standard deviation ($\bar{x}\pm s$). A one-way ANOVA was used for comparison between groups that conformed to a normal distribution, the rank-sum test was used for comparison between groups that did not conform to a normal distribution, and *X*^2^ test was used for count data. *p* < 0.05 was considered to be statistically significant.

### 2.5. rs1801133-PPARG Signaling Pathway for H-type Hypertension

Assisted by Elsevier Pathway Studio (version 12.4.0.3: database of functional relationships and pathways of mammalian proteins; Elsevier), an rs1801133-PPARG signaling pathway was constructed to explore the connection between rs1801133 mutation, PPARG, and their potential roles in the pathology and treatment of hypertension and hyperhomocysteinemia. The entities within the pathways include the two main components of enalapril maleate and folic acid tablets (folate and enalapril), C677T polymorphism (rs1801133) in the MTHFR gene, two diseases (hyperhomocysteinemia and hypertension), the protein PPARG, and the small molecule homocysteine. The relationship between the entities within the signaling pathway was supported by results published in previous studies, which covers the full PubMed abstracts and over 6.9M Elsevier and third-party full-text articles. The direction, polarity, and reliability of the relationships were manually checked as a quality control process. Following a similar approach, we also compiled the pathway to explore the influence of aging on H-type hypertension and its treatment.

## 3. Results

### 3.1. Patient Baseline Characteristics

The baseline characteristics of patients are presented in Table 1. A total of 203 patients diagnosed with H-type hypertension were included in this study, of which 114 were women and 89 were men. According to their MTHFR genotype, all the patients were divided into three groups, including 55 patients in the CC group, 96 in CT, and 52 in TT, respectively. CT type accounts for 47.29% of the total H hypertension patients, indicating that most patients have at least a partial mutation (*p* value < 0.05).

As shown in Table 1, the average ages of the CC, CT, and TT groups were 59.2 ± 13.5 years, 59.0 ± 14.0 years, and 53.2 ± 12.6 years, without significant differences among the three groups (*p* value > 0.05) as a whole. However, the average age of the CT group and CC group was significantly higher than that of the TT group (*p* value < 0.05), which indicated that the onset age of H-type hypertension patients might vary according to different rs1801133 phenotypes. For gender and ethnicity, no significant difference was found among the three groups (*p* value > 0.05). In addition, the blood pressure, blood lipids, blood glucose, and atherosclerotic changes were measured and compared among three genotype groups, with no significant difference detected (*p* value > 0.05; see Table 2). These findings suggested that ethnicity, gender, and the blood indicators (blood glucose, CHOL, and TG) of H-type hypertension patients may not associate with rs1801133 mutation.

### 3.2. The Relationship between the rs1801133 Polymorphism and Hcy Levels

The relationship between rs1801133 polymorphism and Hcy levels in the H-type hypertension patients was analyzed. The Hcy levels were measured for patients before and after treatment using EMFAT. As shown in Table 2, before treatment, the levels of Hcy in the CC group, CT group, and TT group were 14.26 ± 4.06, 13.27 ± 2.98, and 25.00 ± 18.07, respectively, showing significant association with rs1801133 polymorphism (*X*^2^ = 6.109, *p* = 0.005). The level of Hcy in the TT group was significantly higher than that of the CC group and the CT group (*p* value < 0.05). These findings indicated that rs1801133 polymorphism was significantly related to the elevated Hcy levels in H-type hypertension patients.

All the patients in three different groups were treated with oral EMFAT (10 mg) once a day. After three-month treatment, the levels of Hcy in the CC group, CT group, and TT group were 11.52 ± 4.58, 11.55 ± 2.40, and 18.22 ± 7.73, which were significantly lower than those before treatment (*p* value < 0.05). To note, the treatment effect by using EMFAT was similar for all three groups in terms of decreasing the Hcy levels (*p* = 0.36). In addition, the between-group Hcy level differences remained high after the treatment (*p* < 0.001), which highlighted the impact of rs1801133 mutation on Hcy levels in H-type hypertension patients.

### 3.3. rs1801133-PPARG Signaling Pathway for H-type Hypertension

**Figure 1:**
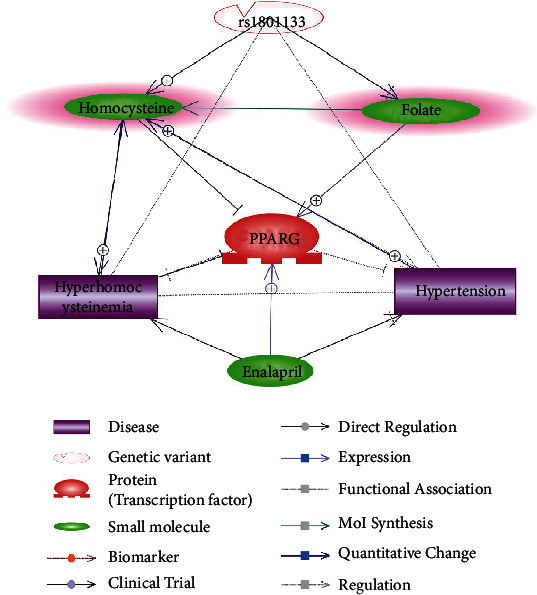
MTHFR-C677T-PPARG signaling pathway for H-type hypertension.

The signaling pathway constructed through literature data analysis is presented in Figure 1. The composed pathway demonstrated the possible linkage among rs1801133 mutation, PPARG, homocysteine, folate, enalapril, hyperhomocysteinemia, and hypertension. Specifically, it showed that rs1801133 mutation could promote plasma homocysteine and inhibit folate, which led to decreased PPARG expression and increased risk of hypertension and hyperhomocysteinemia. Meanwhile, treatment using folate and enalapril increased the mRNA level and expression of PPARG, which contributed to the decrease of plasma homocysteine levels and blood pressure. The pathway suggested that both rs1801133 mutation and PPARG could play a role in the pathological development of H-type hypertension. Moreover, it might add new insights into understanding the treatment effect of EMFAT on H-type hypertension.

### 3.4. Role of Aging in H-type Hypertension

**Figure 2:**
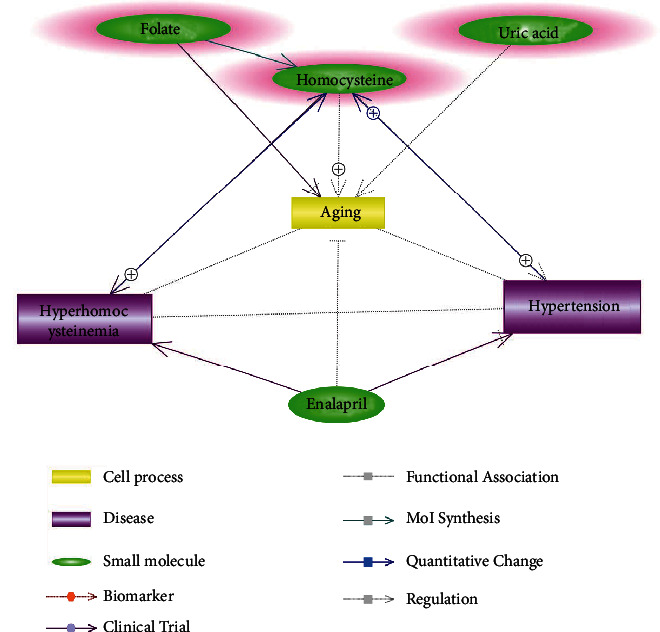
Pathway showing the role of aging in H-type hypertension and its treatment using enalapril maleate and folic acid tables.

As we noted that patients in the TT group were much younger than that in the CT and CC groups, we composed the pathway to explore the role of aging in the pathogenesis of H-type hypertension and its treatment. As shown in Figure 2, aging is a risk factor for increased homocysteine levels [20], decreased uric acid [21], and folate deficiency [22], which increases the risk of hypertension and hyperhomocysteinemia. The fact that younger patients in the TT group presented similar blood pressure as elder patients (TC and CC groups) might highlight the influence of higher Hcy levels on blood pressure.

Interestingly, Griffin et al.'s study using the rat model showed that enalapril not only prevented the increase in blood pressure but even abrogated the blood pressure increases with aging [23], which partially explains the treatment effect of H-type hypertension using EMFAT.

## 4. Discussion

MTHFR is the rate-limiting enzyme in the methyl cycle, which includes the conversion of homocysteine into methionine. The C677C to T mutation in the catalytic region of the MTHFR gene might induce valine to replace alanine, leading to the enzyme's thermolability and the inhibition of MTHFR activity, decreasing the transformation from 5,10-methyltetrahydrofolate to 5-methyltetrahydrofolate [24, 25]. As a result, the level of plasma Hcy increases and causes specific pathological changes, including vascular endothelial damage, vascular smooth muscle proliferation, inhibition of nitric oxide production, and the development of hypertension [26–29].

In this study, the polymorphism of rs1801133 was analyzed in 203 H-type hypertension patients from the Hulunbuir region. The results indicated that 27.09% of patients were wild-type homozygous (CC), 47.29% were heterozygous (CT), and the remaining individuals (25.62%) were mutant allele homozygous (TT), indicating that most patients have heterozygous (CT) (*p* value < 0.05) (Table 1). To note, the distribution of CT genotype was similar to that in the previous study (49.95%) [30], which supported the validity of the clinical data employed in this study.

There was no difference in the distribution of rs1801133 genotypes based on sex and ethnicity (*p* value > 0.05), which was consistent with the report from the majority of previous studies [11]. However, we noted that patients in TT genotype were significantly younger than patients in CC or CT genotype group (*p* value < 0.05). Aging is the most common risk factor for the development of hypertension [31] and hyperhomocysteinemia [20], which was also implicated in the pathway presented in Figure 2. In other words, older rather than younger people are more susceptive to the risk of hypertension. Clinical data analysis showed that patients within TT genotype group presented significantly higher Hcy levels (*χ*^2^ = 6.109, *p* < 0.005 before treatment; *χ*^2^ = 7.87, *p* < 0.001 after treatment). Taken together, these results suggested that patients with TT genotype of rs1801133 were at a higher risk of developing hypertension at a younger age, which might be associated with the increased Hcy level. Our results suggested that the rs1801133 TT genotype was associated with H-type hypertension.

The literature-based pathway analysis (Figure 1) supported our clinical data analysis results that rs1801133 could lead to increased Hcy levels and decreased folic acid levels [30] and, therefore, could be a potential risk factor for H-type hypertension. Moreover, the influence of rs1801133 mutation on H-type hypertension may be partially through the inhibition of PPARG. On the one hand, homocysteine was an inhibitor of PPARG [19], which got promoted by rs1801133 TT mutation [30]. On the other hand, rs1801133 TT mutation decreases plasma folic acid [30], which was a promoter of PPARG [32]. Therefore, the mutation of rs1801133 could act as an inhibitor of PPARG. However, PPARG has been shown to play a protective role in hypertension [15] and has been demonstrated to lower plasma homocysteine [17]. In this sense, the downregulation of PPARG could partially explain the effect of rs1801133 mutation on the development of H-type hypertension.

Moreover, pathway analysis also showed that both major components of EMFAT, enalapril and folate, have been shown to unregulate PPARG [32, 33]. Therefore, as an inhibitor of hypertension and hyperhomocysteinemia, PPARG might also be involved in the treatment of H-type hypertension using EMFAT (Figure 1).

This study has several limitations that need future work. First, the clinical data were collected from the Hulunbuir region with a limited sample size (203 patients), which may not represent H-type hypertension patients in general. Our results should be tested using more clinical data of larger size from different population regions. Second, the pathways built in this study were based on previous studies with different backgrounds. The pathways should be validated using data from the direct H-type hypertension study.

## 5. Conclusion

Our results support that rs1801133 mutation could play a role in the development of H-type hypertension, which may be partially through the downregulation of PPARG. Moreover, PPARG may also be involved in the treatment of H-type hypertension using EMFAT.

## Figures and Tables

**Table 1:** Patient baseline characters.

Group	Number of cases (%)	Age	Gender		Ethnicity		
			F	M	Han	Mongolian	Others
CC	55 (27.09)	59.2 ± 13.5	26	29	43	9	3
CT	96 (47.29)	59.0 ± 14.0	62	34	73	17	6
TT	52 (25.62)	53.2 ± 12.6	26	26	45	5	2

**Table 2:** Comparison of measured indicators among the three groups.

	CC	CT	TT	*χ* ^2^	*p*
Blood glucose (mmol/L)	5.50 ± 0.44	6.11 ± 2.99	5.84 ± 1.14	0.61	0.873
CHOL (mmol/L)	4.87 ± 0.73	5.28 ± 1.00	5.49 ± 0.96	0.23	0.956
TG (mmol/L)	2.08 ± 1.42	1.47 ± 0.48	1.80 ± 0.59	1.28	0.298
SBP (mmHg)	150.59 ± 16.12	152.77 ± 15.41	151.60 ± 20.13	1.25	0.339
DBP (mmHg)	86.64 ± 16.84	82.00 ± 10.94	87.60 ± 9.63	1.37	0.279
Pre-Hcy (*μ*mol/L)	14.26 ± 4.06	13.27 ± 2.98	25.00 ± 18.07	6.11	0.005
Post-Hcy (*μ*mol/L)	11.52 ± 4.58	11.55 ± 2.40	18.22 ± 7.73	7.87	0.001
The changes of Hcy level	2.740 ± 2.40	1.71 ± 3.87	6.780 ± 14.20	86.00	0.360
The incidence rate of atherosclerotic changes in carotid color Doppler ultrasound (%)	27.3	54.5	30	2.994	0.224

## Data Availability

The data in our study are available from the corresponding author upon reasonable request.
